# Interleukin-4 and Interleukin-13 Gene Polymorphisms in Children With Idiopathic Nephrotic Syndrome

**DOI:** 10.3389/fped.2020.591349

**Published:** 2020-11-19

**Authors:** Maysoun Al Rushood, Amal A. Al-Eisa, Mohammad Z. Haider

**Affiliations:** Department of Pediatrics, Faculty of Medicine, Health Sciences Center, Kuwait University, Kuwait City, Kuwait

**Keywords:** interleukin-4, interleukin-13, nephrotic syndrome, gene polymorphisms, cytokine, steroids

## Abstract

**Background:** Idiopathic Nephrotic syndrome (INS) is an immune-mediated disease in which a number of cytokines, including IL-4 and IL-13, have been implicated in the pathogenesis. Cytokine gene polymorphisms might affect their levels and activity. Therefore, may affect INS susceptibility and response to treatment. The aim of the study was to determine the association of IL-4 and IL-13 gene polymorphisms and INS susceptibility and their effects on steroid responsiveness in children.

**Methods:** The polymorphisms in IL-4 and IL-13 genes were detected by PCR-RFLP in 155 INS patients and 64 controls.

**Results:** A total of 132 steroid-sensitive (SS) and 23 steroid resistance (SR) INS patients; mean age 7.3 ± 4.0 years, were included. Male: Female ratio was 2:1. No significant statistical differences were detected in the frequency of CC, CT, and TT genotypes of IL-4 gene compared to controls (*P* = 0.57, 0.61, and 1.00, respectively). There was no significant difference in the T and C-allele frequencies, in SS and SR subgroups. Analysis of IL-13 gene polymorphism also did not show significant statistical differences in the frequency of QQ, RQ, and RR genotypes compared to controls (*P* = 0.74, 1.00, and 0.68, respectively). No significant difference was found in the Q and R-allele frequency. However, the heterozygous RQ genotype of the IL13 gene was significantly higher in SS INS patients compared to the SR INS cases (*P* = 0.04).

**Conclusion:** Our findings did not show an association between IL-4 and IL-13 gene polymorphisms and INS susceptibility. However, IL-13 RQ genotype was expressed more in children with INS who are steroid sensitive.

## Introduction

Idiopathic nephrotic syndrome (INS) is the most frequent form of nephrotic syndrome in children, representing more than 90% of cases between the age of 1 to 10 years (1). Immunological disturbances, especially T cell imbalance, have been implicated in the pathogenesis of the disease (1, 2). This association was suggested by the response to immunosuppressive treatment and the association between the disease with atopy and elevated serum IgE levels (1, 2).

T helper cells (Th2) with its signature cytokines such as IL-4 and IL-13 have been studied as potential important role players in the pathogenesis of the INS (3, 4). IL-4 is a Th2 cytokine produced by basophils, mast cells and activated TH2 cells (5), and plays an essential role in IgE regulation, Th2 differentiation, Th1 inhibition and induction and maintenance of allergy (6, 7). It might also act as an anti-tumor and an anti-inflammatory agent (8, 9).

On the other hand, IL-13 is an immunoregulatory protein produced by T-cells subsets, basophils, eosinophils and mast cells. It is believed to be an important mediator in allergic disease as its levels and genetic polymorphism variants have been shown to be associated with elevated serum IgE levels in atopic and asthmatic patients of different racial backgrounds (10–13).

Both IL-4 and IL-13 genes are located in a region of 140 kb on chromosome 5q31-33 that codes for a cluster of Th2 cytokines (10, 14). They share a common IL-4 receptor α chain (IL4 RA) in the multimeric IL-4 and IL-13 receptor complexes (15). Therefore, they share many biological activities that affect clinical conditions.

Gene Polymorphisms in the regulatory regions of these cytokines can influence the amount of cytokine produced as well as their biological activity and potency at their receptor sites (14). Cytokine gene polymorphism including that of IL-4 and IL-13 in nephrotic syndrome of children with an Arab race has not been explored previously. The aim of this study is to determine any association between IL-4 and cytokine IL-13 gene polymorphisms with the susceptibility to INS in Kuwaiti children and their effects on response to steroid treatment.

## Methods

Children less than 12 years of age with a confirmed diagnosis of Idiopathic nephrotic syndrome (INS) were included in this study. All had INS with a clinical and/or histopathological diagnosis of MCNS. Subjects were evaluated at a specialized pediatric nephrology clinic in Mubarak Al-Kabir University Hospital during the period 2012–2018. INS was defined as the presence of generalized edema, nephrotic- range proteinuria, hypoalbuminemia with or without hyperlipidemia (16). A total of 155 children were included. Patients were subdivided according to their response to steroids into steroid sensitive (SS) and steroid resistant (SR). Steroid responsiveness was defined as the disappearance of proteinuria (negative to trace in a urine dipstick for 3 consecutive days, or a urine protein/creatinine level of <0.2) within the first 4-week course of full dose prednisolone therapy (60 mg/M^2^/day). Steroid resistance was defined as the persistence of proteinuria after a 4-week course of full dose prednisolone. Patients with poor compliance and not on regular follow-up were excluded as well as those with infrequent relapses. All SSNS patients were in full remission at the time of the study.

All patients underwent a thorough physical examination by an experienced pediatrician. A total of 64 age and gender- matched healthy children of the same ethnic background, were included as controls. All were examined by a senior pediatrician to exclude any renal disease. Controls were selected from patients visiting Hospital's Emergency Department for minor illnesses such as upper respiratory tract infection or acute gastroenteritis.

In all the study subjects (patients and controls), tests for blood urea, serum creatinine, total protein, albumin level and a complete lipid profile were carried out. Urine protein: creatinine ratio was also determined for all the study subjects.

At the end of the study, all SSNS patients had normal serum creatinine. Sixty-five percent of the patients were in remission for more than 6 months, while 35% continued to relapse, but less frequently, despite receiving second-line immunosuppressive treatment. For SSNS patients, renal biopsy was performed for steroid dependent patients and for those with frequent relapses (> 6 relapses/year) before initiating cytotoxic therapy.

Genetic study was done in 12 SRNS patients and they were NPHS1 negative. The genetic study for the rest of the SRNS patients was not available as it should be sent outside the country.

### Genotyping of IL4 and IL13 Gene Polymorphisms

DNA was isolated from the peripheral leukocytes using a standard method (17). The genotypes were determined by PCR-RFLP (polymerase chain reaction-restriction fragment length polymorphism) method using the primers listed below (18, 19):

### IL-4 Gene C590T Polymorphism

Forward primer: 5′-ACTAGGCTCACCTGATACG-3′

Reverse primer: 5′-GTTGTAATGCAGTCCTCCTG-3′

The PCR reactions were carried out in a total volume of 25 μl containing 100 ng of genomic DNA, 10 pmoles of each primer, 2 mM MgCl_2_, 0.2 mM deoxynucleotides (dNTPs), 1 × buffer, and 2U of Taq DNA polymerase. The amplification was performed for 35 cycles with an annealing temperature of 58°C for 1 min for the IL-4 gene C590T polymorphism. The C—->T transition at codon 590 of the IL-4 gene abolished a restriction site for *Bsm*F1 in the T-allele. The polymorphism was identified by *Bsm*F1 restriction endonuclease digestion of the PCR-amplified fragment. The cleavage products were analyzed by 2% agarose gel electrophoresis and visualized under UV light after staining with ethidium bromide. The product sizes were 192 and 60 bp for the C-allele and 252 bp for the T-allele, respectively.

### IL-13 Gene Polymorphism

The IL-13 gene polymorphisms R130Q (rs20541) and IL-13 gene promoter polymorphism (C- 1112T) was determined by PCR-RFLP methods described earlier (18, 19). The PCR primer sequences used were:

Sense primer: 5′-CTTCCGTGAGGACTGAATGAGACGGTC-3′

Antisense primer: 5′-GCAAATAATGATGCTTTCGAAGTTTCAGTGGA-3′

For the R130Q polymorphism, amplification was carried out after denaturation at 94^0^C for 5 min followed by 35 cycles of 94^0^C for 45 s, 67^0^C for 45 s and 72^0^C for 30 s followed by an extension at 72^0^C for 5 min. The PCR products were cleaved with restriction enzyme *Nla*IV (0.5 U) at 37^0^C for 3 h and analyzed by agarose gel electrophoresis as described earlier. The expected cleavage products were 210 and 26 bp when a normal R130- genotype is present and in the case of mutant −130Q genotype the expected product size 178 bp, 32 and 26 bp, respectively.

### Statistical Analysis

The genotypes were determined by gene counting and the data was analyzed using the Chi-squared test and the Fisher's exact test. Odds Ratio (OR) was calculated with 95% Confidence Interval (CI). *P*-values of 0.05 or less were considered as statistically significant.

Ethical approval was obtained from the Health Sciences Center Committee for the Protection of Human Subjects in Research, Kuwait University, as well as, Ministry of Health Ethics Committee. An informed consent was obtained from care givers of both patients and controls as per guidelines of the Committees.

## Results

The study included a total of 155 Kuwaiti children with INS. Of these, 132 were steroid-sensitive (SS) and 23 were steroid resistant (SR). The mean age in the INS patient group was 7.6 ± 4.3 years and Male: Female ratio was 2:1. The clinical and biochemical characteristics of INS patients and controls have been presented in Table 1. A total of 83 patients had history of atopy (53.5%), with some having more than one form of atopy. Asthma was reported in 53 patients (34%), eczema in 46 patients (27%), allergic conjunctivitis in 11 (7%), and allergic rhinosinusitis in 7 patients (4.5%). Food allergy was not reported in any patient. Atopy was not reported in any of the controls.

**Table 1:** Clinical and laboratory data of Kuwaiti children with Idiopathic Nephrotic Syndrome (INS) and the controls.

		**INS patients**	**Controls**	**Normal**
**Range**		**(*n* = 155)**	**(*n* = 64)**	
Mean age (years)		7.6 ± 4.3	7.1 ± 3.7	
Gender	Male	104	42	
	Female	51	22	
Atopy (*n*)		83 (53.5%)	0 (0%)	
Mean serum creatinine (μmol/L)		47 ± 7	56 ±*8*	(15–88)
Mean serum protein (g/L)		50 ± 2	73 ± 2	(68–80)
Mean serum albumin (g/L)		21 ± 3	34 ± 3	(30–40)
Mean serum cholesterol (mmol/L)		6.5 ± 0.4	3.2 ± 0.6	(3.1–5.2)
Mean UP: Cr ratio^*^ (mg/mg)		2.8 ± 0.7	0.02 ± 0.05	(≤ 0.2)

* *UP, Urine Protein; Cr, Creatinine*.

Genotype analysis of IL-4 gene polymorphism was inconclusive in 2 INS patients of the SS subgroup which were excluded from the statistical analysis for IL-4 gene polymorphism. From a total of 153 INS subjects and 64 controls, the CC genotype of IL-4 gene polymorphism was detected in 64% of the INS patients compared to 69.5% in the controls (*P* = 0.57). The heterozygous CT genotype was detected in 30% of INS patients compared to 25.5% in the controls (*P* = 0.61). The TT-genotype was detected in 6% of INS patients and in 5% of the controls (*P* = 1.00). The C-allele frequency in homozygous and heterozygous forms was found in 94% of INS patients compared to 95% of the controls (*P* = 1.00). The T-allele frequency in homozygous and heterozygous forms was found in 35.7% of INS patients compared to 30.5% of the controls (*P* = 0.57). Table 2 summarized the gene and allele frequency of IL-4 gene polymorphism in both patients and controls. No significant difference was detected in any of the genotype frequencies between the SS and SR sub-groups when compared with each other or when compared to the controls (Table 3).

**Table 2:** Interlukin-4 gene polymorphism in Kuwaiti children with INS and the controls.

**Genotype**	**INS (%)**	**Controls (%)**	**Odds ratio**	**95% confidence**	***P*-values**
	***n* = 153**	**(*n* = 64)**	**(OR)**	**Interval (CI)**	
CC	99 (64.71)	44 (68.75)	0.83	0.45–1.56	0.68
CT	45 (29.41)	17 (26.56)	1.15	0.59–2.22	0.79
TT	9 (5.88)	3 (4.69)	1.27	0.33–4.86	1.00
C allele	243/306	105/128	0.84	0.49–1.44	0.62
T allele	63/306	23/128	1.18	0.69–2.01	0.62

**Table 3:** Comparison of IL-4 gene polymorphism genotype and allele frequencies between steroid sensitive (SS) and steroid resistant (SR) Kuwaiti INS patients.

**Genotype**	**SS (%)**	**SR (%)**	**Odds ratio**	**95% confidence**	***P*-values**
	***n* = 130**	**(*n* = 23)**	**(OR)**	**interval (CI)**	
CC	83 (63.85)	16 (69.57)	0.77	0.29–2.01	0.64
CT	40 (30.77)	5 (21.74)	1.60	0.55–4.61	0.46
TT	7 (5.38)	2 (8.69)	0.59	0.12–3.08	0.63
C allele	226/260	37/46	1.62	0.72–3.65	0.25
T allele	54/260	9/46	1.08	0.49–2.37	1.00

In the case of IL-13 gene polymorphism, genotyping was conclusive for all the 155 INS patients and 64 controls and the results have been presented in Table 4. The QQ genotype of IL-13 gene polymorphism was detected in 70.3% of the INS patients compared to 73.4% of the controls (*P* = 0.74). The heterozygous RQ genotype was detected in 25.8% of INS patients compared 28% of the controls (*P* = 1.0). The RR-genotype was detected in 3.9% of INS patients and 1.6% of the controls (*P* = 0.68). The Q-allele frequency in homozygous and heterozygous forms was found in 83.2% of INS patients compared to 86% of the controls (*P* = 0.57). The R-allele frequency in homozygous and heterozygous forms was found in 16.7% of INS patients compared to 14% of the controls (*P* = 0.57). When the genotype frequencies were compared between the SS and SR sub-groups, only the RQ genotype showed a statistically significant association with steroid sensitivity (*P* = 0.04; Table 5).

**Table 4:** Genotype and allele frequencies of Interleukin-13 gene polymorphism in Kuwaiti children with INS and controls.

**Genotype**	**INS (%)**	**Controls (%)**	**Odds ratio**	**95% confidence**	***P*-values**
	***n* = 155**	**(*n* = 64)**	**(OR)**	**interval (CI)**	
QQ	109 (70.32)	47 (73.44)	0.86	0.45–1.65	0.74
RQ	40 (25.81)	16 (25.0)	1.04	0.53–2.04	1.00
RR	6 (3.87)	1 (1.56)	2.54	0.29–21.52	0.68
Q allele	258/310	110/128	0.81	0.45–1.45	0.57
R allele	52/310	18/128	1.23	0.69–2.20	0.57

**Table 5:** Comparison of IL-13 gene polymorphism genotype and allele frequencies between steroid sensitive (SS) and steroid resistant (SR) Kuwaiti INS patients.

**Genotype**	**SS (%)**	**SR (%)**	**Odds ratio**	**95% confidence**	***P*-values**
	***n* = 132**	**(*n* = 23)**	**(OR)**	**interval (CI)**	
QQ	90 (68.18)	19 (82.60)	0.45	0.14–1.41	0.22
RQ	38 (28.79)	2 (8.70)	4.25	0.95–19.00	0.04
RR	4 (3.03)	2 (8.70)	0.33	0.05–1.90	0.22
Q allele	218/264	40/46	0.71	0.28–1.78	0.53
R allele	46/264	6/46	1.41	0.56–3.51	0.53

The data of SS patients and SR patients were studied separately and compared to controls. When IL-4 gene polymorphisms data of SR and SS patients were compared to controls separately, no significant differences in the genotype or allele frequencies were detected, as shown in Tables 6, 7.

**Table 6:** Comparison of IL-4 gene polymorphism genotype and allele frequencies between Steroid Sensitive (SS) patients and controls.

**Genotype**	**SS (%)**	**Controls (%)**	**Odds ratio**	**95% confidence**	***P*-values**
	***n* = 130**	**(*n* = 64)**	**(OR)**	**interval (CI)**	
CC	83 (63.85)	44 (68.75)	0.80	0.42–1.52	0.61
CT	40 (30.77)	17 (26.56)	1.23	0.63–2.40	0.66
TT	7 (5.38)	3 (4.69)	1.16	0.29–4.63	1.00
C allele	226/260	105/128	1.46	0.82–2.60	0.26
T allele	54/260	23/128	1.20	0.70–2.06	0.61

**Table 7:** Comparison of IL-4 gene polymorphism genotype and allele frequencies between Steroid Resistant (SR) patients and controls.

**Genotype**	**SR (%)**	**Controls (%)**	**Odds ratio**	**95% confidence**	***P*-values**
	**(*n* = 23)**	**(*n* = 64)**	**(OR)**	**interval (CI)**	
CC	16 (69.57)	44 (68.75)	1.04	0.37–2.92	1.00
CT	5 (21.74)	17 (26.56)	0.77	0.25–2.39	0.78
TT	2 (8.69)	3 (4.69)	1.94	0.30–12.40	0.60
C allele	37/46	105/128	0.90	0.38–2.12	0.83
T allele	9/46	23/128	1.11	0.47–2.62	0.83

Similarly, IL-13 gene polymorphisms data of SR and SS patients were compared to controls separately. No significant differences in the genotype or allele frequencies were detected, when each sub-group compared to controls, as shown in Tables 8, 9.

**Table 8:** Comparison of IL-13 gene polymorphism genotype and allele frequencies between Steroid Sensitive (SS) patients and controls.

**Genotype**	**SS (%)**	**Controls (%)**	**Odds ratio**	**95% confidence**	***P*-values**
	***n* = 132**	**(*n* = 64)**	**(OR)**	**interval (CI)**	
QQ	90 (68.18)	47 (73.44)	0.78	0.40–1.51	0.56
RQ	38 (28.79)	16 (25.0)	1.21	0.61–2.39	0.70
RR	4 (3.03)	1 (1.56)	1.97	0.22–17.99	1.00
Q allele	218/264	110/128	0.78	0.43–1.40	0.48
R allele	46/264	18/128	1.29	0.71–2.33	0.48

**Table 9:** Comparison of IL-13 gene polymorphism genotype and allele frequencies between Steroid Resistant (SR) patients and controls.

**Genotype**	**SR (%)**	**Controls (%)**	**Odds ratio**	**95% confidence**	***P*-values**
	**(*n* = 23)**	**(*n* = 64)**	**(OR)**	**interval (CI)**	
QQ	19 (82.60)	47 (73.44)	1.72	0.51–5.78	0.57
RQ	2 (8.70)	16 (25.0)	0.29	0.06–1.36	0.14
RR	2 (8.70)	1 (1.56)	6.00	0.52–69.62	0.17
Q allele	40/46	110/128	1.09	0.40–2.94	1.00
R allele	6/46	18/128	0.92	0.34–2.47	1.00

## Discussion

The immunopathogenesis of INS has been studied extensively during the recent years (1, 5, 10). The effects of various cytokines on the glomerular basement membrane in the kidneys is widely supported by many studies on different populations (1, 5, 10). IL-4 and IL-13 are two important mediators which have recently attracted attention in studies exploring the pathogenesis of INS. As many INS patients commonly develop atopy, and IL-4 and IL-13 are the major mediators involved in that process, it was not surprising to incriminate these two cytokines in the immune-pathogenesis of the INS. Several previous reports have demonstrated that both these cytokines share a number of biological activities including IgE isotope switching, CD23 induction and stimulation of eosinophils activity as they both share a common IL-4 receptor alpha chain (IL-4RA) in the multicentric IL-4 and IL-13 receptor complexes (5, 6, 8, 15).

In the present study, we did not find an association between IL-4 or IL-13 gene polymorphism and predisposition to INS in Kuwaiti Arab children.

Many previous studies reported conflicting results (2, 10, 20). When our findings were compared with other populations worldwide, our results were consistent with data obtained from Caucasians in the UK where IL-4 gene polymorphism was studied in 100 patients and 63 controls with no significant difference between allele distribution in INS patients and the controls (2). However, Kobayashi et al. (20) studied IL-4 gene polymorphism in 58 Japanese children and found that the frequency of the T allele was significantly lower in INS group than the controls. It has been previously reported that T allele of the 590C/T polymorphism was associated with increased IL-4 gene promoter activity (21, 22). Another study carried out in India on 150 children with INS, demonstrated that IL-4-C590T polymorphism may influence the prognosis and the clinical course of the disease (23).

Our study did not show any association between IL-13 gene polymorphism and susceptibility of children with an Arab racial background to INS. However, the RQ genotype was associated with steroid sensitivity.

These findings were consistent with studies reported in different other populations worldwide. Data in both Asian and Caucasian populations suggest that there is no association of IL-13 gene polymorphism with susceptibility to minimal change nephrotic syndrome (10, 24, 25). Similar results have been reported from British INS patients (24) as well as on German INS patients (25). In Singapore Asian children also, no significant association of IL-13 gene polymorphism with INS was detected (10).

The lack of association of both 1L-4 as well as IL-13 gene polymorphisms and INS reported in our study as well as other studies seems to be unexpectedly surprising knowing the previously documented effects of both these cytokines in kidneys. Many previous *in vivo* as well as *in vitro* studies had supported the important roles of both cytokines and their receptors in the pathogenesis of INS. Podocytes in the glomerular basement membranes in kidneys of express receptors for both interleukins IL-4 and IL-13 (26, 27). These cytokines have been shown *in vitro* to have direct effects on podocyte mediator production and barrier function (26–28). A mechanism whereby excess IL-13 might induce nephrotic syndrome is illustrated by changes in podocyte protein trafficking and proteolytic enzyme secretion seen when these cells are incubated with IL-4 or IL-13 *in vitro* (29).

Another intriguing animal study directly addressed the role of IL-13 by overexpressing it in the rats and reported that an MCN-like nephropathy was induced, with changes in podocyte structure and gene expression similar to those seen in human diseases (30).

Moreover, Van den Berg showed the presence of IL-13 receptors on glomerular epithelial cells, the stimulation of which, resulted in a decrease in the transepithelial electrical resistance suggesting a possible direct effect of this cytokine on the podocytes and its function in preserving circulatory albumin and preventing albuminuria, which is the hallmark of INS (27). It has been reported that children with nephrotic syndrome who had higher initial serum IgE levels, had higher relapsing rates, longer duration of steroid therapy before remission and higher serum IL-4 and IL-5 levels (31).

A dysfunctional glomerular filtration barrier characterizes nephrotic syndrome, the pathophysiology of which is highly complex. T cells, mainly Th2 and Th17, play an important role. The upregulation of specific cytokines (IL-4, IL-5, IL-9, and IL-13) was previously shown to promote the development of NS (32). Therefore, studying the gene polymorphisms of these cytokines is essential.

Our study might be limited by a relatively small sample size. It would be prudent to have the serum cytokine levels for correlation. Despite being a single center study, ours is the focal center for nephrotic syndrome in the entire country where all the complex cases are referred so it covers the whole country. Although we studied patients with the same ethnic background, it is actually a strength of our study because this type of genetic association has not been performed in any population in the Gulf Region. We studied the homozygous and the heterozygous genotypes. The allele frequencies in the homozygous and heterozygous forms were included.

In conclusion, we report no association between IL-4 and IL-13 gene polymorphisms and the predisposition of INS in Kuwaiti Arab children. Of note, IL-13 RQ genotype was expressed more in children with INS who are steroid sensitive. Further larger studies, involving samples of different ethnic backgrounds, are warranted to determine the genetic predisposition of INS, as well as, steroid responsiveness in children.

## Data Availability Statement

The raw data supporting the conclusions of this article will be made available by the authors, upon request.

## Ethics Statement

The studies involving human participants were reviewed and approved by Health Sciences Center committee for the protection of human subjects in research, Kuwait University, as well as, Ministry of Health ethics committee, Kuwait. Written informed consent to participate in this study was provided by the participants' legal guardian, patients and controls, as per the committees recommendations.

## Author Contributions

MA and AA-E: substantial contributions to conception and design. AA-E: acquisition of data. MH: gene studies and data analysis. MA and AA-E: interpretation of the results. MA and AA-E: drafting the article. AA-E and MH: revising it critically for important intellectual content. All authors: final approval of the version to be published.

## Conflict of Interest

The authors declare that the research was conducted in the absence of any commercial or financial relationships that could be construed as a potential conflict of interest.
